# Gut microbiome modulates efficacy of immune checkpoint inhibitors

**DOI:** 10.1186/s13045-018-0592-6

**Published:** 2018-03-27

**Authors:** Ming Yi, Shengnan Yu, Shuang Qin, Qian Liu, Hanxiao Xu, Weiheng Zhao, Qian Chu, Kongming Wu

**Affiliations:** Department of Oncology, Tongji Hospital of Tongji Medical College, Huazhong University of Science and Technology, Wuhan, 430030 China

**Keywords:** Gut microbiome, Immunotherapy, PD-1/PD-L1, CTLA-4, ICIs resistance

## Abstract

Immune checkpoint inhibitors (ICIs) therapy is a novel strategy for cancer treatments in recent years. However, it was observed that most patients treated with ICIs could not get benefit from the therapy, which led to the limitation of clinical application. Motivated by potent and durable efficacy of ICIs, oncologists endeavor to explore the mechanisms of resistance to ICIs and increase the drug sensitivity. It is known that heterogeneity of gut microbiome in populations may result in different outcomes of therapy. In xenograft model, bacteria in gut have been proved as a crucial factor regulating immunotherapy efficacy. And the similar phenomenon was obtained in patients. In this review, we summarized relevant advancements about gut microbiome and ICIs. Furthermore, we focused on modulatory function of gut microbiome in ICIs therapy and possible antitumor mechanism of specific commensals in ICIs treatment. We propose that gut microbiome is an important predictive factor, and manipulation of gut microbiome is feasible to elevate response rate in ICIs therapy.

## Background

Various bacteria populating in mammal gastrointestinal tract are an indispensable part in intestine ecosystem and play a pivotal role in gut barrier [1]. Commensal bacteria have been showed to regulate host immune system through the crosstalk with host intestinal epithelial cells and lymphatic cells [2]. In addition, the bacterial metabolism is another factor influencing the host immune homeostasis [2]. The existence of commensal bacteria not only protects host from infection of pathogens, but also plays a crucial role in some diseases, including inflammatory bowel diseases, type 1 diabetes mellitus, adiposity, metabolic and cardiovascular disorders, dyslipidemia, asthma, allergy, as well as cancers [3–10]. The diversity and abundance of the commensal bacteria could be evaluated by several identification methods (16S rRNA gene sequencing, metagenomics analysis, and qPCR technology) [11, 12]. Analysis revealed that loss of diversity and shift in gut microbiome composition were related to immune-mediated diseases. Manipulating gut microbiome may provide a promising strategy for treatment.

Notably, this influence on host immune system even affects the efficacy of some agents, although the exact mechanism is unknown. Immune checkpoint inhibitors (ICIs), known as the novel immunotherapy agents, take significant and durable curative effects on advanced hematological and solid malignancies [13–15]. Simultaneously blocking two signaling pathways of ICIs, including programmed cell death protein 1/programmed cell death 1 ligand 1 (PD-1/PD-L1) and cytotoxic T-lymphocyte antigen-4 (CTLA-4), may enhance antitumor effects remarkably in spite of increased side reactions [16–18].

Nevertheless, the majority of patients shows primary or acquired resistance during treatment (up to 60–70% in melanoma and even higher rate in other cancers), which limits the clinical application of ICIs [19]. Because of the limited choice for patients with refractory tumors, the appearance of ICIs brings a new hope for the treatment of tumor even though the dissatisfactory effective rate. How to identify the patients who are most likely to benefit from ICIs is a hot topic [20, 21]. It is also crucial to avoid resistance and enhance the efficacy of ICIs treatment. Recent studies revealed the association between gut microbiome and ICIs efficacy, which provided us a new perspective on immunotherapy interference.

## The role of gut microbiome in physiology and pathology process

Symbiotic commensals in human gastrointestinal tract play a vital role in sustaining host homeostasis and health. Symbiotic commensals participate in many physiological functions. As a part of digestive system, symbiotic commensals promote process of saccharide digestion and absorption by transforming polysaccharides into disaccharides and monosaccharides [22, 23]. By fermentation of dietary fiber, some commensal bacteria, such as *Lachnospiraceae*, *Ruminococcaceae*, and *Butyrate producing bacterium L2-21*, upregulate butyrate level in lumen which is responsible for intestinal epithelial cells renewal. Specific commensal bacteria are also correlated with outcompeting pathogens and the synthesis of essential vitamins for human, such as vitamin B and vitamin K [24, 25]. Therefore, dysregulated gut microbiome is involved in the progression of many diseases, including cancers [22]. It is acknowledged that specific gut bacteria contribute to the tumorigenesis through multiple regulatory manners. Pathogens which may induce oncogenesis could be antagonized by the normal commensal floras [22]. Besides, specific bacteria mediate the conversion from the primary bile acids to secondary bile acids. Deoxycholic acid, belonging to the secondary bile, produces free radicals, damages DNA, and promotes oncogenesis in liver, esophagus, and colon [22]. Pathogenicity islands on bacterial chromosome are gene clusters which associate with bacterial virulence, and they are generally believed to participate in the initiation of colorectal cancer [26]. Metalloproteinase, the product of pathogenicity island in *Enterotoxigenic Bacteroides fragilis*, undermines the integrity of gut barrier and increases the chance of immune tissue’s exposure to bacteria and metabolites, which in turn activate inflammation responses and increase colorectal cancer risk [22].

Notably, resident commensal bacteria modulate host immune system by cross-talk with epithelial cells and lymphoid structures [27]. It has been confirmed that gut commensals regulate composition of lymphocytes subsets in secondary immune organs, such as Peyer’s patches [28]. Taking segmented filamentous bacteria (SFB) as an example, the overrepresentation of SFB in mice leads to increased level of Th17, Th1, IFN-γ, and IL-17, thereby stimulating immune response [27]. Moreover, through penetrating the mucus layer, SFB interact with epithelial cells thereby influencing epithelium signal pathways, upregulating antimicrobial proteins, and promoting Th17 cells polarization [27]. Some bacteria, such as species of *Clostridiales*, suppress immune response by inducing Tregs differentiation and IL-10 production in intestine and extra-intestine [29]. Apart from influencing local immunity, commensal bacteria regulate systemic immunity. Polysaccharide (PSA) produced by *B. fragilis* has the ability to correct immune deficiencies (Th1/Th2 imbalance and CD4^+^ T cells deficiency) in germ-free mice [30]. *E. hirae* induces pathogenic Th17 (pTh17) cells response and increases cytotoxic T cells/Tregs ratio in extra-intestinal tissue, while *B. intestinihominis* enhances systemic Tc1 and Th1 response [31]. However, at the same time, gut microbiome is shaped by host immunity as well [32]. In mouse model, the most bacterial abundance is downregulated by innate and adaptive immune response [32]. Even the morphology of some bacteria could be influenced by host immunity which hampers the interaction between bacteria and epithelial cells in turn [32].

Due to the advancement of sequencing technology, especially the appearance of Next-Generation Sequencing (NGS) technology, it is available to analyze composition of microbiota. Bacterial 16S rRNA sequencing and metagenomic shotgun sequencing have been widely applied for taxonomic assignment. Bacterial 16S rRNA sequencing provides a convenient access to analyze the microbiota [33]. Because of the species specificity of bacterial 16S rRNA, taxonomic identification could be carried out by comparison with the known 16S rRNA databases [33]. However, the main flaw of 16S rRNA sequencing is the limitation of database. Therefore, it would be difficult to identify unknown bacteria [33]. The metagenomic shotgun sequencing overcomes the disadvantage of 16S rRNA sequencing by analyzing the whole genomic context. And metagenomic sequencing could be used in taxonomic assignment as well as functional analysis of microbial community [34].

## The antitumor roles of ICIs

ICIs, including CTLA-4 and PD-1/PD-L1, are the monoclonal antibodies to specific receptors on cell membrane and aim to block the signaling pathways which negatively modulate the immune system. ICIs restore the exhausted T cells and activate the immune system to promote destruction of tumor cells through blocking related signaling pathways mentioned above. PD-1 is the most important immunotherapy target, expressed on tumor infiltrating lymphocytes (TILs) and other immune cells [35]. PD-1 is a transmembrane receptor, composed of extracellular domain, transmembrane domain, and intracellular tail [36]. PD-L1/PD-L2 are ligands of PD-1, contributing to maintain tissue homeostasis in the context of infection [36]. PD-L1 is constitutively expressed on the membrane of antigen-presenting cell (APC), and it is upregulated in the condition of APC activation [37]. Besides, PD-L1 is also widely expressed in lymphatic and non-lymphatic tissues [38]. On the contrary, PD-L2 is predominantly found in APCs. Immune receptor tyrosine-based inhibitory motif (ITIM) and immune receptor tyrosine-based switch motif (ITSM), as the crucial structures in PD-1 pathway, recruit Src homology 2 domain containing phosphatases 1/2 (SHP1/2) and mediate the inhibitory function [39]. In tumor microenvironment, overexpression of PD-L1 is stimulated by IFN-γ or oncogenic driver events [36]. PD-1 binds to PD-L1 and subsequently inhibits PI3K-AKT and Ras-Raf-MEK-ERK signaling pathways [36]. The intracellular downstream signals of PD-1/PD-L1, act as a brake on the activation of effector T cells, suppress proliferation and differentiation of effector T cells, and impair neoantigen presentation process [38, 40–42]. The administration of PD1/PD-L1 blockade could recover T cells from exhausted status and normalized tumor site immune response [43].

CTLA-4 receptor is another target for immunotherapy, similarly to PD-1/PD-L1 signaling pathway, negatively regulating immune system. CTLA-4 is constitutively expressed in CD4^+^ CD25^+^ Foxp3^+^ regulatory T cells, and it is upregulated transiently in activated conventional T cells [44]. Sharing two ligands with co-stimulation receptor CD28, CTLA-4 has higher affinity and avidity for CD80 (B7.1) and CD86 (B7.2) than CD28 [45]. Through competitively binding to these ligands, CTLA-4 acts as an antagonist of CD28 and leads to the impairment of T cells response [45, 46]. Besides, during the process of CTLA-4 internalization, CTLA-4 undergoes endocytosis accompanied with the ligand [47]. CTLA-4 is recycled back to cell membrane while the ligand is degraded, which requires more ligands expressed on the surface of APCs to compensate for the depletion [47]. In tumor microenvironment, increased Tregs result in depletion of CD80 and CD86, so it is hard for CD28 to maintain normal immune co-stimulation process. Increased activation threshold of T cells as well as impeded proliferation of tumor-specific T cells contributes to T cells anergy [48]. Apart from acting as the competitive antagonist for T cell activation, CTLA-4 is generally believed to dampen immune response by mediating cellular signaling pathways in T cell. CTLA-4 binds to phosphatidylinositol 3-kinase (PI3K) by Val-Tyr-Val-Lys-Met (YVKM) motif, SHP2, and protein phosphatase 2A (PP2A) [49]. And SHP2 and PP2A are related with production of negative signals [49]. Furthermore, CTLA-4 could block ZAP-70 microcluster formation which is essential to signal transmission of T cell receptor (TCR) [49]. By manners mentioned above, CTLA-4 inhibits IL-2 production and induces T cell exhaustion [49]. ICIs increase the CD80 and CD86 on membrane of APCs, and effective antigen presentation enhances the antitumor ability. Since the first ICI (ipilimumab) was approved for advanced melanoma treatment by FDA in 2011, many drugs have gone through phase 3 trial and been applied in clinical fields, including anti-PD-1 monoclonal antibodies (nivolumab, pembrolizumab)/anti-PD-L1 monoclonal antibody (atezolizumab) and anti-CTLA-4 monoclonal antibody (ipilimumab) [50–52]. By mechanism distinguished from conventional treatments, ICIs show unprecedented therapeutic effect on some refractory tumors. However, the resistance rate in patients is too high to choose the ICIs as the first line agents (except for NSCLC and melanoma) in the tumor treatment guideline [53, 54].

## ICIs resistance

The clinical trial Keynote 006 (NCT01866319), involving 843 patients with advanced melanoma, showed that the patients receiving pembrolizumab treatment had response rates ranging from 33.7% (10 mg/kg every 2 weeks) to 32.9% (10 mg/kg every 3 weeks), while patients receiving ipilimumab (3 mg/kg every 3 weeks) had a worse response rate of 11.9% [55]. Besides, after follow-up 7.9 months treatment, 10.6, 3.3, 12.1% responding patients in aforementioned groups showed acquired resistance, respectively [55]. This study reflected a severe issue in the clinical application of ICIs: primary resistance and acquired resistance. Here, we took PD-1 blockade resistance as an example to discuss in detail. According to the results in vitro and in vivo, the resistance to PD-1/PD-L1 is related to many factors. (A) The tumor mutational burden and immunogenicity [19]. Primary resistance is prevalent in patients with some poor antigenicity tumors, including prostate and pancreas tumor [19]. Besides, immunoediting during tumor development is associated with immune escape, resulting in the acquired resistance [56]. (B) Upregulated other immune checkpoints as compensatory bypass tracks [57]. T-cell immunoglobulin mucin-3 (TIM-3) is another immune checkpoint co-expressed with PD-1, especially in exhausted T cells [58, 59]. Accordingly, during the treatment of PD-1 blockade, patients showed acquired resistance accompanied with increased expression of TIM-3. (C) Extracellular inhibitory metabolites in local microenvironment [60]. Indoleamine 2, 3-dioxygenase (IDO) is produced by tumor cells and lymphatic cells in melanoma patients, and is regarded as a biomarker of progression and invasion [61]. Adenosine is another local extracellular metabolite mediating T cells dysfunction [62]. Accumulating adenosine in tumor microenvironment correlates to poor clinical outcome as well as worse antitumor efficacy through adenosine receptor and adenosinergic pathway [63]. A_2A_ receptor and adenosinergic pathway which consists of CD39 and CD73, participate in angiogenesis, metastasis, and immune suppression [64–66]. Moreover, apoptosis of Tregs resulting from oxidative stress leads to amplified immune suppression by releasing adenosine, which is related to PD-1 blockade resistance [67].

## Studies on the role of gut microbiome in ICIs efficacy

Distinguished from cytotoxic therapies, ICIs mediates tumor regression via enhanced host immune activation. Some studies revealed the shift in gut microbiome composition influencing ICIs efficacy. As early as 2015, researchers noticed the relationship between gut symbiotic bacteria and PD-1 blockade. Ayelet Sivan et al. explored the influence of *Bifidobacterium* on PD-1 blockade treatment, using two strains mouse models (JAX/TAC) bearing B16.SIY melanoma [68]. The subcutaneous tumor issues showed different invasion abilities influenced by immune responses. More proportion of intratumoral CD8^+^ T cells and more potent tumor-specific immune response were observed in JAX, and the difference was abrogated by cohousing. Besides, researchers found that transferring fecal microbiome from JAX to TAC could elevate specific tumor lymphocytes and suppress tumor growth. Interestingly, in TAC, just fecal microbiome transfer from JAX could inhibit tumor growth in the same degree with PD-1 blockade treatment, and it had synergetic effect with PD-1 blockade treatment in promoting tumor regression [68]. Gut microbiome analysis at genus level revealed that *Bifidobacterium* abundance was related to tumor specific immune cytotoxicity, and the abundance increased over 400-fold after fecal microbiome transplantation in TAC [68]. By 16S rRNA gene sequencing, *Bifidobacterium* operational taxonomic units (OTUs) were identified to be similar with *Bifidobacterium breve*, *Bifidobacterium longum*, and *Bifidobacterium adolescentis* in 99% identity (Table 1). Treating by commercial cocktail of *Bifidobacterium* with or without PD-1 blockade, both showed significant antitumor effect, compared with *Bifidobacterium*-treated group. Researchers attributed the enhanced antitumor effect to increased IFN-γ production, maturation activation, and shift in the function of dendritic cells (DCs) [68].

**Table 1:** Modulatory function of gut microbiome in ICIs therapy

Bacteria	Model	Modulatory function of gut microbiome in ICIs therapy	Author/year	Ref.
*Akkermansiacea muciniphila*	Human/mouse	a) Synergistic effect with PD-1 blockade b) Increasing TCM,CD4/Foxp3 ratio in tumor bed and IL-12 production c) Increasing IFN-γ production	Bertrand Routy 2017	[12]
*Alistipes indistinctus*	Human/mouse	Restoring antitumor efficacy of PD-1 blockade	Bertrand Routy 2017	[12]
*Bacteroides fragilis*	Mouse	a) Immunostimulation induced by CTLA-4 blockade b) Inducing activation of Th1 cell and promoting maturation of DC in tumor bed	Marie Vétizou 2015	[70]
*Bacteroides thetaiotaomicron*	Mouse	Promoting Th1 immune response and anti-tumor effect of CTLA-4 blockade	Marie Vétizou 2015	[70]
*Bifidobacterium adolescentis*	Human	a) Related with enhanced efficacy of PD-1 blockade b) Leading to decreased peripherally derived Treg	Matson V 2018	[69]
*Bifidobacterium breve* *Bifidobacterium longum*	Mouse	a) Stimulating DCs directly, inducing DCs maturation and cytokine secretion b) Anti-tumor function and synergistic effect with PD-1 blockade	Ayelet Sivan 2015	[68]
*Bifidobacterium longum*	Human	overrepresentation in R undergoing PD-1 blockade	Matson V 2018	[69]
*Blautia obeum*	Human	Related with compromised efficacy of PD-1 blockade	Matson V 2018	[69]
*Burkholderia cepacia*	Mouse	Contributing to tumor control and promoting Th1 immune response	Marie Vétizou 2015	[70]
*Butyrate producing bacterium L2-21*	Human	a) Prolonging PFS/OS and enhancing CTLA-4 blockade efficacy b) Promoting activation of tolerogenic macrophage and DC c) Inducing activation of Treg	N. Chaput 2017	[72]
*Collinsella aerofaciens*	Human	a) Related with enhanced efficacy of PD-1 blockade b) Leading to decreased peripherally derived Treg	Matson V 2018	[69]
*Enterococcus faecium*	Human	a) Related with enhanced efficacy of PD-1 blockade b) Leading to decreased peripherally derived Treg	Matson V 2018	[69]
*Enterococcus hirae*	Human/mouse	a) Synergistic effect with PD-1 blockade combined with *Akkermansia muciniphila* b) Elevates TCM combined with *Akkermansia muciniphila* c) Increasing production of IFN-γ	Bertrand Routy 2017	[12]
*Faecalibacterium prausnitzii L2-6*	Human	a) Prolonging PFS/OS and enhancing CTLA-4 blockade efficacy b) Associated with induction of Treg in gut	N. Chaput 2017	[72]
*Gemmiger formicilis*	Human	a) Prolonging PFS/OS and enhancing CTLA-4 blockade efficacy b) Elevating colitis risk	N. Chaput 2017	[72]
*Klebsiella pneumonia*	Human	Related with enhanced efficacy of PD-1 blockade	Matson V 2018	[69]
*Parabacteroides merdae*	Human	a) Related with enhanced efficacy of PD-1 blockade b) Leading to decreased peripherally derived Treg	Matson V 2018	[69]
*Roseburia intestinalis*	Human	Related with compromised efficacy of PD-1 blockade	Matson V 2018	[69]
*Ruminococcaceae*	Human/mouse	a) Related with enhanced efficacy of PD-1 blockade b) Elevating level of effector T cells in peripheral blood and TIL.	Gopalakrishnan V 2017	[11]
*Veillonella parvula*	Human	Related with enhanced efficacy of PD-1 blockade	Matson V 2018	[69]

Abbreviation: *R* responding individuals, *NR* non-responding individuals, *PFS* progression-free survival, *OS* overall survival, *TILs* tumor infiltrating lymphocytes, *DC* dendritic cell

Then, two studies involving some advanced tumor patients for further exploration of relationship between gut microbiome and PD-1 blockade were carried out subsequently in 2017. Gopalakrishnan V et al. analyzed differences of gut microbiome diversity and composition between responding individuals (R) and non-responding individuals (NR) [11]. Through metagenomics analysis, researchers analyzed 43 patients’ fecal samples, including 30 R and 13 NR, drawing a conclusion that there was a significant clustering effect of microbiome in each group, and α-diversity was significantly higher in N than NR [11]. Analysis of OTUs revealed that patients enriched in *Clostridiales/Ruminococcaceae* were prone to respond to PD-1 blockade effectively, contrary to ones enriched in *Bacteroidales* (Table 1). Then from the results of metagenomics analysis at all levels, *Faecalibacterium* genus (one genus of *Ruminococcaceae* family, *Clostridiales* order) caught researchers’ attention [11]*.* Patients with high abundance of *Faecalibacterium* had longer PFS (*p* = 0.03) and advantage in hazard ratio (HR = 2.92, 95% CI = 1.08–7.89) in comparison with patients with low abundance of *Faecalibacterium* [11]. Besides, level of tumor infiltrating CD8^+^ T cells was positively related to abundance of *Faecalibacterium* genus, contrary to *Bacteroidales* order [11]. In peripheral blood, patients with overrepresentation of *Faecalibacterium*, *Clostridiales*, and *Ruminococcaceae* had more effector T cells, while patients with overrepresentation of *Bacteroidales* had more Tregs and myeloid-derived suppressor cells [11]. Multiple immunohistochemistry showed more immune markers in patients enriched in *Faecalibacterium* [11]*.* The fecal microbiome transplantation in mice supported the conclusions aforementioned [11].

Meanwhile, Bertrand Routy et al. compared outcomes of patients undergoing PD-1 blockade with or without utilizing antibiotics. The antibiotics-treated group had shorter PFS and OS undergoing PD-1 blockade alone or combined with CTLA-4 blockade [12]. Besides, antibiotics treatment was an independent factor of PD-1 blockade. And researchers observed overrepresentation of *Akkermansia muciniphila* was the most significant factor related to potent response (*p* = 0.004, in overall; *p* = 0.003, excluding antibiotics-treated individuals) and better clinical outcome (Table 1) [12]. Analyzing the relationship between clinical outcome and immune response showed that reaction of Th1/Tc1 to *Akkermansia muciniphila* and reaction of Tc1 to *Enterococcus hirae* by IFN-γ secretion were correlated to clinical outcome [12]. Trial in mouse model verified the conclusion: broad-spectrum antibiotics-treated or germ-free mice receiving fecal microbiome transplantation from non-responding individuals showed significant compromised antitumor effect of PD-1 blockade or PD-1 combined with CTLA-4 blockade [12]. Interestingly, the antibiotics-treated mice restored PD-1 blockade efficacy by recolonization of *Akkermansia muciniphila* with or without *Enterococcus hirae*. Oral gavage of *Akkermansia muciniphila* and *Enterococcus hirae* resulted in abundant production of IL-12 and CCR9^+^CXCR3^+^ central memory T cells, increased secretion of IFN-γ, and higher CD4/Foxp3 ratio in tumor bed [12]. Almost simultaneously, Matson V et al. analyzed fecal samples from metastatic melanoma patients and concluded some commensal bacterial species which potentiated tumor-specific immunity and enhanced efficacy of PD-1 blockade treatment [69]. And the mice which were subjected to fecal material transplantation from R tended to have a slower tumor growth rate and better treatment effect of PD-1 blockade [69].

Apart from PD-1/PD-L1, CTLA-4 is another hot research issue. Marie Vétizou et al. conducted a trial to study the influence of *Bacteroidales* on CTLA-4 blockade efficacy [70]. In MCA205 sarcomas mouse model, specific pathogen-free (SPF) mice had the advantage over germ-free (GF) and broad-spectrum antibiotics-treated mice in treatment efficacy [70]. In turn, perturbation of commensal floras resulting from CTLA-4 blockade was observed. Elevated abundance of some specific species (*B. thetaiotaomicron* and *B. uniformis*) was accompanied with the decrease of *Bacteroidales* and *Burkholderiales* (Table 1) [70]. Notably, *Bacteroides fragilis*, confirmed as immune modulating bacteria species, showed no significant changes during treatment [70, 71]. Besides, with administration of some specific species, the resistance in GF and broad-spectrum antibiotics-treated mice was overcame, and transferring *B. fragilis*-specific memory Th1 could restore antitumor effect partially [70]. Through transplanting fecal microbiome from melanoma patients, researchers observed that *B. fragilis* overrepresentation was relevant to tumor regression [70]. Interestingly, the utilization of vancomycin was confirmed to enhance the efficacy of ipilimumab, but alleviated adverse effect did not parallel with the elevated treatment effect. It was assumed that vancomycin indirectly promoted *Bacteroidales* overrepresentation by inhibiting the proliferation of *Clostridiales* [70].

However, in 2017, another trial focusing on the relationship between gut microbiome at baseline and clinical benefit was conducted in metastatic melanoma individuals, and the result was conflicted with conclusion of Marie Vétizou. Inconsistent with the conclusion of trial in mouse models, the study found that both *B. fragilis* and *B. thetaiotaomicron* were in low proportion at baseline and *Bacteroidales* overrepresentation impeded antitumor function of CTLA-4 overall (Table 1) [72]. Besides, some *Firmicutes*, including *Faecalibacterium* genus, *butyrate producing bacterium*, and *G. formicilis* were found to be related with higher response rate and better clinical outcome (longer PFS and OS) [72]. Contrary to the trials mentioned above, the antibiotics treatment did not influence the dominant microbiota composition or bacterial species which possibly influence the efficacy [72]. Given the compromised ICIs efficacy caused by utilization of antibiotics in previous studies, this conflicting result was worthwhile to explore further [11, 12, 73]. The discrepancies between trials were attributed to some factors. Firstly, the bias existing in fecal microbiome transplantation and the differences between mouse and human model limits the conclusion extrapolation [72]. Apart from this, in mouse experiment, it is hard to exclude other bacteria species interfering results because of the limitation of qPCR analysis targeting some specific species of *Bacteroidales* [72]. Notably, contrary to conclusion of Marie Vétizou et al., no significant shift in gut microbiome composition resulting from CTLA-4 blockade was observed except for patients with therapy-induced colitis [72]. Given that Mao K et al. noticed the state of gut microbiota was influenced by innate and adaptive immune response, it is reasonable to speculate that unleashed T cell would reshape gut microbial communities, change the quantity and proportion of some specific bacterial species, and even affect the morphology and function of bacteria [32]. And further studies should be carried out to evaluate the effect of ICIs on gut microbiota.

## Synergetic antitumor mechanism of specific commensals in ICIs treatment

**Fig. 1:**
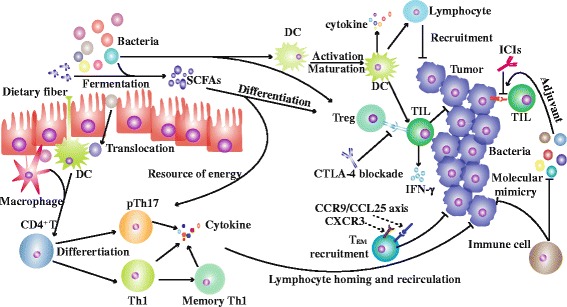
The potential mechanism of gut microbiome regulating ICIs efficacy. Firstly, the abundance of CTLA-4 on Tregs is upregulated by some bacteria and metabolites at baseline, which increases sensitivity to CTLA-4 blockade. Secondly, gut microbiota enhances the function of DCs. For example, *Bifidobacterium* promotes DCs maturation and decreases activation threshold, elevates recruitment and function of T cells by interaction with DCs. Thirdly, administration of *Akkermansia muciniphila* and *Enterococcus hirae* results in elevated CD4^+^ TCM in tumor bed. Fourthly, commensal bacteria are sensed by APCs, inducing pTh17 and Th1 differentiation, which influence tumor immunity by lymphocyte homing and recirculation. Fifthly, SCFAs are utilized by immune cells and gut epithelial cells as source of energy. Lastly, molecule mimicry theory and adjuvant effect participate in immune response

Gut microbiome has been verified to participate in oncogenesis as well as immune surveillance suppressing malignant transformation [74–77]. By influencing gut immune homeostasis and immune tune of second immune organs, specific commensals have synergetic functions with treatments, including immunotherapy, radiotherapy, chemotherapy, and surgery effect [78–87]. Here, we highlighted possible manners gut microbiome involving in the ICIs efficacy (Fig. 1).

### Interaction with Tregs

Owing to the predominant expression of CTLA-4 in Tregs, the status of Tregs at baseline is pivotal to determine CTLA-4 blockade efficacy, which is distinguished from PD-1 blockade. Tregs play a crucial role in modulating immune response and correlate to the failure of immune co-stimulation process. Some specific bacteria species and metabolites have been confirmed to participate in Tregs differentiation. Bacteria including *Faecalibacterium prausnitzii* and *Clostridia* class induce Tregs differentiation and suppress inflammation [88, 89]. Gut microbiome derived short-chain fatty acids (SCFAs), such as butyrate and propionate, promote Tregs differentiation and change the size of Treg pool by elevating acetylation level of histone H3 in Foxp3 promoter region and conserved non-coding region [88, 90]. Besides, researchers found patients enriched in *Faecalibacterium* and other species of *Firmicutes* had lower proportion of systematic inflammation lymphocytes at baseline. On the contrary, patients enriched in *Bacteroides* had increased systematic inflammation biomarkers in peripheral blood at baseline, accompanied with decreased colitis risk and clinical benefit [72]. Presumably, anti-inflammatory bacteria and metabolites induce Tregs differentiation and promote Tregs proliferation, resulting in higher CTLA-4 level [72]. Increased CTLA-4 level leads to increased sensitivity to CTLA-4 blockade by abrogating immune suppress in gut and tumor tissue probably, which is related with colitis incidence and potent efficacy [72]. The conclusion was partly proved by Krista Dubin’s study in 2016, which showed that *Bacteroides* led to the decreased colitis incidence risk [91].

Increased CTLA-4 level at baseline helps tumor cells escape from immune surveillance, but it increases the sensitivity to CTLA-4 blockade simultaneously. This factor is more crucial for CTLA-4 blockade in comparison with PD-1 blockade, because of greater depletion of Tregs resulting from CTLA-4 blockade [92]. PD-1 blockade plays a role by unleashing T cells, so other factors promoting activation of T cells contribute to tumor regression as well. However, patients undergoing CTLA-4 blockade could get benefits from enhanced activation of T cells theoretically.

### Enhanced function of DCs

Enhanced function of DCs is generally believed as an important manner to promote T cell activation. *Bifidobacterium* has been confirmed to upregulate gene transcription of DCs, associated with interaction of cytokines, maturation of DCs, and activation of lymphocytes [68]. The shift results in the upregulation of lymphocytes recruitment, more potent neoantigen presentation process and cytokines production [68]. Besides, the threshold for DCs’ activation is downregulated, which means less antigen concentration acquired for T cells priming. At low antigen concentration, DCs could upregulate IFN-γ production and promote T cells proliferation in priming process [68]. Increased intratumoral specific CD8^+^ T cells and enhanced lymphocytes function have synergetic effects with ICIs, contributing to tumor regression.

### Memory T cells

Colonization of *Akkermansia muciniphila* and *Enterococcus hirae* in gut is related with emergence of CD4^+^ central memory T cell (T_CM_) in tumor bed, tumor draining lymph code, and mesenteric lymph code [12]. Intriguingly, T_CM_ expresses chemokine receptor CXCR3 and/or CCR9. CXCR3 and CCR9/CCL25 axis have been verified to be related with prolonged PFS and OS in patients with some advanced tumors [93, 94]. CXCR3 is related to recruitment of Th1 cells to inflamed lesions, while CCR9/CCL25 axis is associated with chemotactic migration of T cells, especially in intestine and colon [12]. Presumably, the T cells recruitment increased CD4/Foxp3 ratio in tumor bed.

### Bacteria-specific immune response

ICIs undermine gut immune tolerance, accompanied with response targeting these commensal bacteria. Patients with memory T cells response targeting *Akkermansia muciniphila* and *Enterococcus hirae* are prone to having longer PFS [12]. Though bacteria translocation has not been observed during ICIs treatment, it is still reasonable given the destruction of tolerance of peripheral organs [95, 96]. Intestinal epithelial cell damage during CTLA-4 and PD-1 blockade treatment leads to the loss of integrity of gut barrier. And the translocation of some commensal bacteria such as *Enterococcus hirae*, to secondary immune organs even tumor bed by impaired gut barrier may influence systemic inflammation [31]. Besides, gut microbiome could be sensed by APCs without bacterial translocation as well. Bacteria-specific immune response not only produces inflammation in intestinal mucous, but also promotes differentiation of pTh17 and Th1 in secondary immune organs [31, 78]. Response of memory Th1 and pTh17 to specific bacteria are related to alteration of immune surveillance in tumor microenvironment, by lymphocyte homing and recirculation.

### Other regulatory factors

Except for mechanism mentioned above, some other factors are speculated to involve in ICIs treatment. Some bacterial metabolites, such as SCFAs, can be utilized by intestinal epithelial cells as source of energy [97, 98]. SCFAs prevent autophagy of intestinal epithelial cells and lymphocytes resulting from nutrient starvation [99]. Potential molecular mimicry between commensal bacteria and tumor cells, even though have not been confirmed yet, may influence outcome as well [70]. Besides, some commensals participate in antitumor response by the adjuvant effect [12]. They do not change natural progress of tumor alone, unless in the context of ICIs.

The cancer-immune checkpoint set point model could summarize pathways involving in ICIs treatments, which could be understood as the threshold to overcome for immune response to neoantigen [100]. In this model, cancer-immune checkpoint set point is influenced by immune stimulatory factors, inhibitory factors, and neoantigen presentation process. Some bacteria in the context of ICIs play a role by enhancing tumor-specific immunity, blocking inhibitory signal pathways, and promoting antigen presentation, which could be understood as downregulated cancer-immune checkpoint.

## Conclusions

Cancer immunotherapy includes the use of antibodies, lymphocytes, and cytokines [101, 102]. ICIs are most promising agents of cancer immunotherapy. We retrospect a series of trials in the review to unravel specific commensals related to ICIs efficacy. Researchers utilized mice bearing tumor undergoing fecal microbiome transplantation as model to mimic the alteration process in patients. However, the established tumor in mice by transplanting tumor cells may not represent response in human [80, 103]. Firstly, injection of tumor cells to mice accompanies with tumor cells death, thereby leading to the initial vaccination effect. Secondly, the xenograft model lacks multi-step carcinogenesis and chronic inflammatory in comparison to actual tumor microenvironment [103]. Thirdly, many factors involve in gut microbiota dysbiosis apart from bacteria, such as fungi, virus, and endogenous retrovirus [104–106]. Researchers commonly focus on commensal bacteria and ignore other factors, which could not rule out the interference to conclusion. Finally, it is hard to extrapolate the conclusion in mouse to human. Take the Toll-like receptor (TLR) for example, which is indispensable for innate immunity in commensal bacteria recognition. However, the difference of TLR expression patterns between human and mouse means different lymphocytes involving in immunity [80].

Translation of the findings in mouse model into clinical trial has a long way to go. First of all, identification of bacteria with modulatory ability needs a great quantity of data. Besides, modifying gut microbiota of patient is another obstacle to overcome in clinical trial after identification of favorable microbial communities. Just as widely applied in mouse model experiments, fecal microbiome transplantation is a promising method and other methods are worthwhile to try [107]. Furthermore, detecting the composition of gut microbiota would be helpful for patient selection and efficacy prediction.

## Abbreviation

APC: Antigen-presenting cell
CTLA-4: Cytotoxic T-lymphocyte antigen-4
DC: Dendritic cell
FDA: Food and Drug Administration
GF: Germ-free
ICI: Immune checkpoint inhibitor
IDO: Indoleamine 2, 3-dioxygenase
IFN-γ: Interferon-γ
IL-17: Interleukin-17
ITIM: Immune receptor tyrosine-based inhibitory motif
ITSM: Immuno receptor tyrosine-based switch motif
NGS: Next-Generation Sequencing
NR: Non-responding individuals
OS: Overall survival
OUT: Operational taxonomic unit
PD-1/PD-L1: Programmed cell death protein 1/programmed cell death 1 ligand 1
PFS: Progression-free survival
PI3K: Phosphatidylinositol 3-kinase
PP2A: Protein phosphatase 2A
pTh17: Pathogenic Th17
qPCR: Quantitative polymerase chain reaction
R: Responding individuals
SCFA: Short-chain fatty acid
SFB: Segmented filamentous bacteria
SHP1/2: Src homology 2 domain containing phosphatases 1 and 2
SPF: Specific pathogen-free
Tc1: Cytotoxic T cell 1
TCR: T cell receptor
TIL: Tumor infiltrating lymphocyte
TIM-3: T-cell immunoglobulin mucin-3
TLR: Toll-like receptor
Treg: Regulatory T cell
